# Gradual Degradation of InGaAs LEDs: Impact on Non-Radiative Lifetime and Extraction of Defect Characteristics

**DOI:** 10.3390/ma14051114

**Published:** 2021-02-27

**Authors:** Matteo Buffolo, Alessandro Magri, Carlo De Santi, Gaudenzio Meneghesso, Enrico Zanoni, Matteo Meneghini

**Affiliations:** Department of Information Engineering, University of Padova, 3513 Padova, Italy; alessandro.magri@studenti.unipd.it (A.M.); carlo.desanti@dei.unipd.it (C.D.S.); gauss@dei.unipd.it (G.M.); zanoni@dei.unipd.it (E.Z.); matteo.meneghini@unipd.it (M.M.)

**Keywords:** InGaAs, light-emitting diode, degradation, defects

## Abstract

We present a detailed analysis of the gradual degradation mechanisms of InGaAs Light-Emitting Diodes (LEDs) tuned for optical emission in the 1.45–1.65 μm range. Specifically, we propose a simple and effective methodology for estimating the relative changes in non-radiative lifetime, and a procedure for extracting the properties of defects responsible for Shockley-Read-Hall recombination. By means of a series of accelerated aging experiments, during which we evaluated the variations of the optical and electrical characteristics of three different families of LEDs, we were able to identify the root causes of device degradation. Specifically, the experimental results show that, both for longer stress time at moderate currents or for short-term stress under high injection levels, all the devices are affected: (i) by a partial recovery of the optical emission at the nominal bias current; and (ii) by a decrease in the emission in low-bias regime. This second process was deeply investigated, and was found to be related to the decrease in the non-radiative Shockley-Read-Hall (SRH) lifetime due to the generation/propagation of defects within the active region of the LEDs. Devices tuned for longer-wavelength emission exhibited a second degradation process, which was found to modify the carrier injection dynamics and further speed-up optical degradation in the low bias regime. These processes were ascribed to the effects of a second non-radiative recombination center, whose formation within the active region of the device was induced by the aging procedure. Through mathematical analysis of the degradation data, we could quantify the percentage variation in SRH lifetime, and identify the activation energy of the related defects.

## 1. Introduction

In*_x_*Ga_1−*x*_As alloys are of great interest for the optoelectronics industry, due to their widespread adoption for the manufacturing of short-wavelength infrared (SWIR) sensors, detectors, lasers, and photovoltaic cells [1,2,3,4]. By leveraging the wide tunability of the In_*x*_Ga_1−*x*_As bandgap, detectors and emitters with dominant wavelengths ranging from 870 to 3600 nm can be manufactured. For SWIR applications, a lattice matching condition with commonly available InP substrates can be achieved for a band-to-band material emission at 1.7 μm, with a molar indium fraction *x* = 0.53. The growth of other In*_x_*Ga_1−*x*_As alloys, if not limited to layer thicknesses below the critical value, induces the formation of dislocations and other extended crystalline defects that may be detrimental for the reliable and efficient operation of devices based on this material. The analysis of such defects, as well as the development of mitigation strategies for the epitaxial growth of devices through a metamorphic approach [5,6,7,8], were a focal point of the scientific and industrial research in the 1980s and in the 1990s. The outcome of this research effort enabled the successful introduction to the market of reliable optoelectronic devices to be employed for telecom, solar, and detection applications. Strangely, after that historic moment, a lack of reliability-oriented literature was produced on III-V IR devices; because the major issues related to device defectiveness were solved, the interest of the scientific community moved to device design, or to the analysis of novel material systems. The contemporary need for faster optical interconnections systems [9], and the introduction of new technologies, such as LiDAR [10], that heavily rely on SWIR detectors and sources for their operation, pushed for a re-visitation of the long-term reliability of this kind of devices. In addition, other novel optical sources, such as quantum-dot (QD) lasers epitaxially grown on silicon [11], are adopting InGaAs within their active layer, which further increases the interest toward the reliability of devices featuring this alloy. From that perspective, what was partly missing from the investigations carried out during the latter decades of the 20th century was a more in-depth analysis of the gradual degradation processes limiting the reliability of state-of-the-art devices, and a clear identification of the role that point defects, rather than extended ones, may have had in this process. Results on this may be particularly relevant for the development of advanced sources for silicon photonics based on III-As semiconductors, whose reliability is still under extensive study [12,13,14,15].

To this aim, we decided to preliminarily investigate the degradation mechanisms affecting the stability of the optical performance of three groups of commercially-available SWIR light-emitting diodes, featuring an active layer composed of InGaAs multi-quantum wells (MQWs) tuned for nominal emission wavelengths of 1.45, 1.55 and 1.65 μm. A series of packaged devices was submitted to accelerated life tests, including both current step-stress and constant current aging experiments, during which the variations in the optical and electrical characteristics of the devices were regularly monitored. The experimental results indicate that at low injection levels the optical efficiency of the LEDs is reduced as a consequence of the generation, or propagation, of defects toward the active region of the device. A more in-depth analysis of the degradation data related to the group of devices emitting at longer wavelength allowed us to identify a second degradation process affecting only this specific group of samples. This process, which involved the presence of a new dominant non-radiative recombination center within the active region, was also found to be correlated with the variation of the electrical characteristics, and responsible for the variation in the injection dynamics detected at low bias current. This investigation highlights the role of gradual defect-related degradation in the definition of the reliability of modern InGaAs-based optoelectronic devices. Finally, we proposed a methodology, based on the analysis of the main optical parameters, to identify variations in the non-radiative recombination lifetime, and to describe the activation energy of the related traps.

## 2. Materials and Methods

### 2.1. Experimental Details

The devices investigated within this work are commercially available InGaAs/InP IR LEDs based on an MQW structure, tuned for a nominal emission wavelength ranging from 1450 nm (group A) to 1550 nm (group B) to 1650 nm (group C). The LEDs feature an optical power of about 4.5 mW at the nominal current of 100 mA and at a measuring temperature of T_AMB_ = 25 °C. The 300 × 300 μm^2^ III-V LED chips are mounted on a TO-46 package, featuring a flat window transparent to NIR light, which eased the mounting and testing procedure. The experimental setup was composed of a Thermo Electric Cooler (TEC)-based fixture (model TCLDM9 from Thorlabs, Newton, NJ, USA) driven by a temperature controller (model ITC4005 from Thorlabs, Newton, NJ, USA) and by an HP 4142B parameter analyzer for device bias and electrical measurements (HP is now Keysight Technologies, Santa Rosa, CA, USA). The electrical connection between the parameter analyzer and the fixture was based on a four-wire (Kelvin) configuration, in order to attain a more accurate measurement of the voltage across the device. Optical power was measured by means of an amplified germanium photodiode (model PDA50B-EC from Thorlabs, Newton, NJ, USA) mounted on top of the LED. Finally, the electroluminescence (EL) spectra of the devices were collected by means of a telecom-grade Optical Spectrum Analyzer (OSA) (model AQ6370AD from Yokogawa, Musashino, Tokyo, Japan).

### 2.2. Preliminary Characterization: Electrical Characteristics

We started our investigation by performing a preliminary optical (L–I, electroluminescence (EL)-spectra) and electrical (IV) characterization of all the available samples.

**Figure 1:**
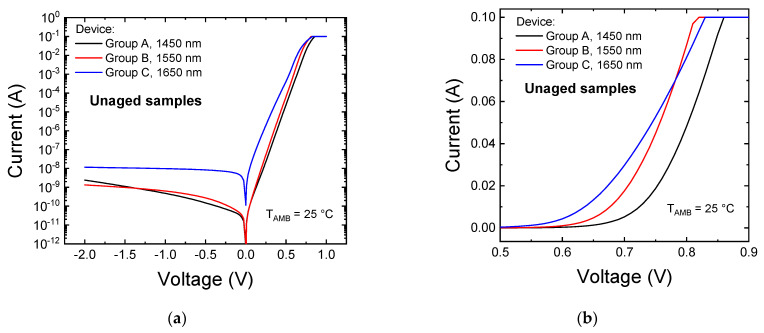

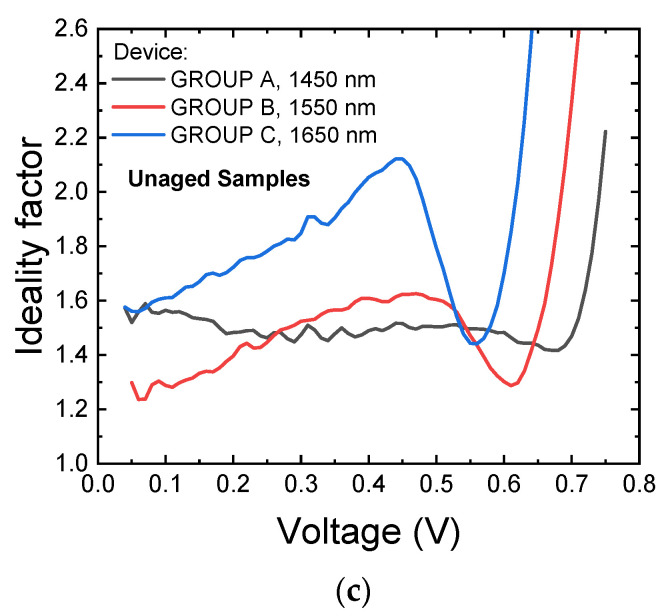
Electrical characteristics of one representative unaged sample for each of the LED groups under investigation, reported (**a**) in semilog scale and (**b**) in linear scale. Plot (**c**) reports the bias-dependent ideality factor calculated for each sample. The measuring temperature during these characterizations was set to T_AMB_ = 25 °C.

The comparison of the electrical characteristics of one representative sample for each group, reported here in Figure 1, shows that group A (1450 nm) and group B (1550 nm) samples exhibited, on average, similar series resistance and forward leakage current values, whereas group C (1650 nm) devices exhibited higher series resistance and leakage current levels, both in reverse and forward bias conditions. Generally speaking, a higher leakage current, if not related to a parallel resistance component, can be associated with an increased concentration of defects in proximity of the junction, possibly indicating an increased defectiveness of group C devices compared to shorter wavelength LEDs. Assuming that tuning of the emission wavelength was achieved through compositional variations rather than through QW width adjustments [16], this may be ascribed to the higher indium content in the semiconductor alloy, that may favor higher defectiveness and the conduction mechanisms, such as trap-assisted tunneling, that typically dominate in the low forward bias regime [17,18]. The higher defectiveness of group C devices was also confirmed by the analysis of the bias-dependent ideality factor, reported here for a representative sample of each group in Figure 1c. The experimental data show that even in an unaged state, LEDs belonging to group C exhibit a higher ideality factor, with values close to 2 at 0.45 V. An ideality factor close to 2 typically indicates a conduction regime dominated by defect-related recombination currents within the space-charge region of the device; therefore, this finding further suggests that group C devices are affected by a higher concentration of defects in the proximity of, or within, their active region.

**Figure 2:**
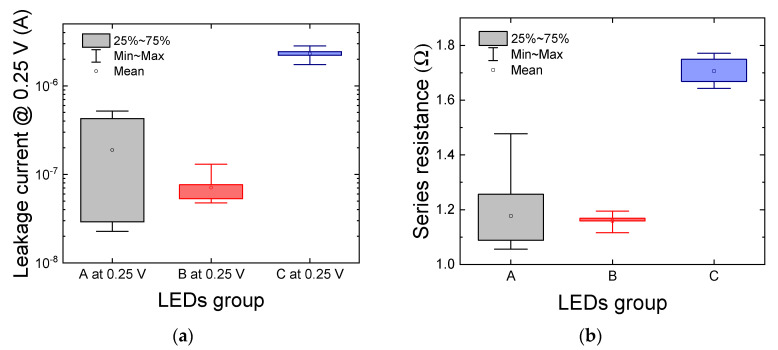
Statistical variation, among a population of 10 devices, of (**a**) the forward leakage current, measured at 0.25 V, and (**b**) of the series resistance of the samples. The box charts show the mean value, minimum and maximum values, and the data range between the 25th and the 75th percentiles. The measuring temperature is T_AMB_ = 25 °C.

The statistical investigation carried out among all the 10 samples available for each LED group confirmed our previous findings, also showing a strong variability of the leakage current (Figure 2a) and series resistance (Figure 2b) of group A devices compared to the other two sets of samples. This may either indicate a problem at process-level, which could have impacted the uniformity of the characteristics of the semiconductor chips spread across single or multiple wafers, or an issue related to the packaging procedure. In this case, a non-optimized die attachment and/or wire-bonding procedure may have led to the observed inconsistency in the series resistance of the devices belonging to group A. It is worth noticing that the forward leakage current of all devices is still limited to the µA range, so the LED’s performance is acceptable for most applications.

### 2.3. Preliminary Characterization: Optical Characteristics

Unaged devices have also been submitted to an extensive optical characterization, which included the evaluation of their EL spectrum, by means of an optical spectrum analyzer, and their light-current-temperature (L–I–T) characteristics.

**Figure 3:**
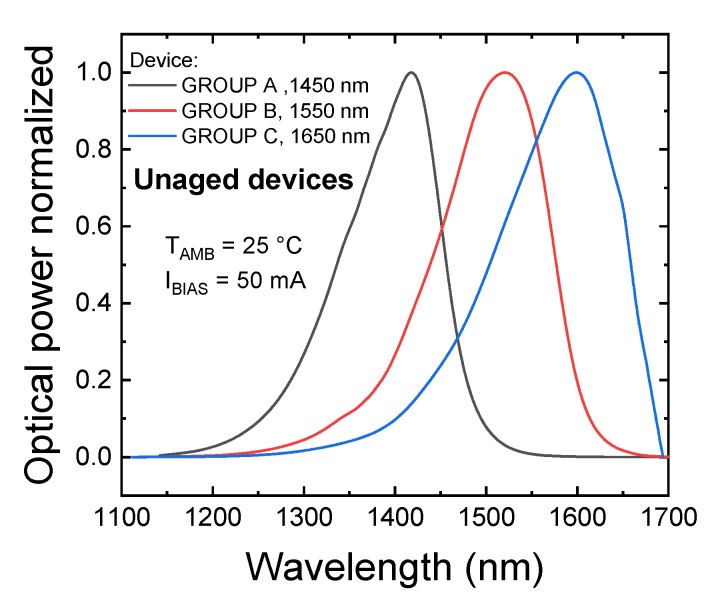
Normalized electroluminescence (EL) spectra, measured at T_AMB_ = 25 °C and I_BIAS_ = 50 mA, of one representative sample for each LED group under investigation.

The spectral analysis, summarized by the normalized OP-λ plot reported in Figure 3, indicates that, at I_BIAS_ = 50 mA and T_AMB_ = 25 °C, the devices have peak emission wavelengths equal to 1419.9 nm (group A), 1519.6 nm (group B) and 1601.4 nm (group C).

**Figure 4:**
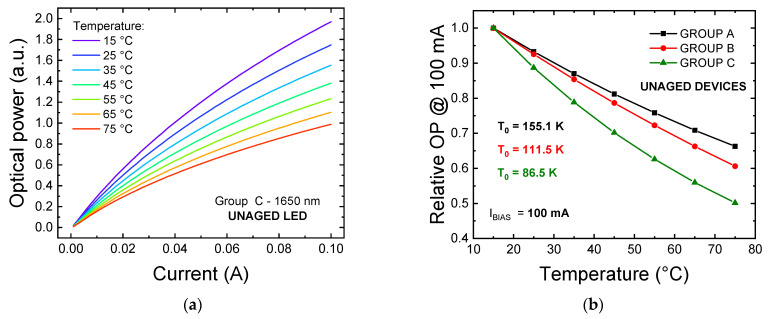
(**a**) L–I–T curves measured on an unaged group C sample; (**b**) normalized thermal drop of the optical power, measured at 100 mA, for one representative samples of each of the three groups of LEDs under investigation.

In terms of L–I–T characteristics, all three groups of devices exhibited a strong drop in optical emission at increasing temperature, as pictured by the L–I–T plot of an unaged group C LED reported in Figure 4a. In order to quantitatively evaluate the magnitude of this process, we calculated the characteristic temperature of the LEDs for each group of devices. This figure of merit [19], typically indicated as T_0_, can be derived from the phenomenological equation that, in most cases, well describes the optical power (OP) trend of a QW LED in function of the measuring temperature, in nominal operating conditions. This relationship is expressed by the following formula:

(1) $$T_{0}=-\frac{T-T_{ref}}{ln\left(\frac{\mathrm{OP}}{{\mathrm{OP}|}_{T_{ref}}}\right)}$$

where T is the measuring temperature, OP is the optical power measured at a temperature equal to T, T_ref_ is the reference temperature with respect to which the drop is calculated, ${\mathrm{OP}|}_{T_{ref}}$ is the magnitude of the optical emission at T_ref_, and T_0_ is the characteristic temperature that we want to extrapolate. In Figure 4b, we report the results of the fitting of the experimental data based on Equation (1), where a T_ref_ of 288.15 K (15 °C) is considered. At the nominal current of 100 mA, the fitting procedure provided values for T_0_ equal to 155.1 K (group A), 111.5 K (group B) and 86.5 K (group C). From Equation (1), we can see that higher values of T_0_ correspond to a lower sensitivity of the optical emission toward temperature variations. Therefore, our experimental results indicate that InGaAs/InP LEDs tuned for emission at longer wavelengths suffer from a more pronounced worsening in optical efficiency as operating temperature rises. In this operating condition, non-radiative recombination processes such as the Shockley-Read-Hall and Auger recombinations are favored [20,21]. In addition, escape processes are also eased by temperature, especially at high injection levels. In order to explain the increased temperature sensitivity at longer wavelengths, we can consider that: (i) group C LEDs may be affected by a worse crystalline quality with respect to groups A and B, as possibly indicated by the higher leakage current of group C devices (Figure 2); ultimately, this means that the increase in SRH recombination with temperature would have a stronger impact on the optical characteristics for group C LEDs, which is compatible with the experimental evidence. (ii) Auger recombination coefficients in III-As quantum wells increase as the bandgap narrows [21]. Finally, (iii) depending on the barrier-to-fundamental QW state energy difference [22], which we can refer to as E_B_-E_0_, the carrier escape may be stronger for MQW structures tuned for emission at longer wavelengths. Validating this hypothesis is outside the scope of this work, and would require additional information regarding the epitaxial structure of the devices. Nevertheless, if we assume the barrier material to be the same for all three groups of devices under test (DUTs), the rate of escape in similar temperature and carrier density conditions would be lower for long-wavelength LEDs, due to the increased quantum well depth.

## 3. Experiments Results and Discussion

After the preliminary characterization of the devices presented in the previous paragraphs, we began our investigation of the degradation processes by performing a current step-stress on one sample for each group. This kind of procedure allowed us to evaluate the short-term impact of a wide range of stress conditions on the optical and electrical characteristics of the devices. The outcome of these experiments was used for the selection of the stress currents that were adopted for the constant-current accelerated aging experiments, needed to properly evaluate the dependencies of the degradation kinetics of the devices.

### 3.1. Current Step-Stress

During a step-stress experiment, the magnitude of an operating parameter, which is known to impact on the degradation of a specific property of a device, is gradually increased until a status of “failure” or of strong degradation is reached. For LEDs, the two main accelerating factors for optical degradation are represented by junction temperature and current-density. With our procedure, we investigated the impact of this latter parameter by performing a series of one hour-long stress steps, starting with a bias current of 20 mA and gradually increasing the stress level by 20 mA steps, up to 1 A. After each step, we performed a complete L–I–T and I–V–T characterization of the device. The stress was performed at an ambient temperature of T_STRESS_ = 45 °C, in order to slightly accelerate the aging process and attain sufficiently high degradation levels in a reasonable amount of time.

**Figure 5:**
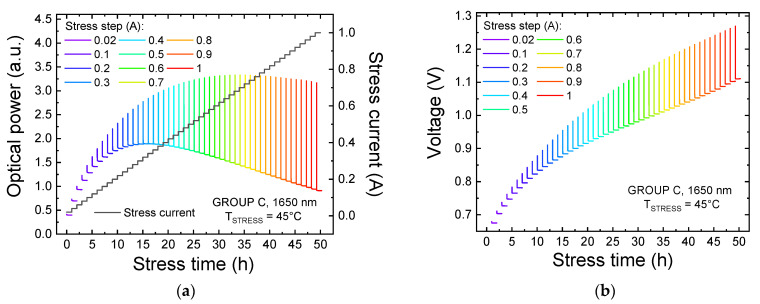
Variation of the optical power (**a**) and of the operating voltage (**b**) of an LED of group C submitted at the current step-stress procedure at T_STRESS_ = 45 °C.

The plots reported in Figure 5a,b show the trends of the optical power and of the LED voltage, respectively, recorded during the entire step-stress procedure, carried out on a group C device. The visible decrease in both operating voltage and OP after the beginning of each stress step is ascribed to the self-heating of the devices, which becomes more prominent as the stress current increases. On the other hand, no relevant time-dependent variation in the emitted OP at high injection levels is visible, possibly indicating that the stress is not strongly degrading the optical performance of the devices at these bias levels.

**Figure 6:**
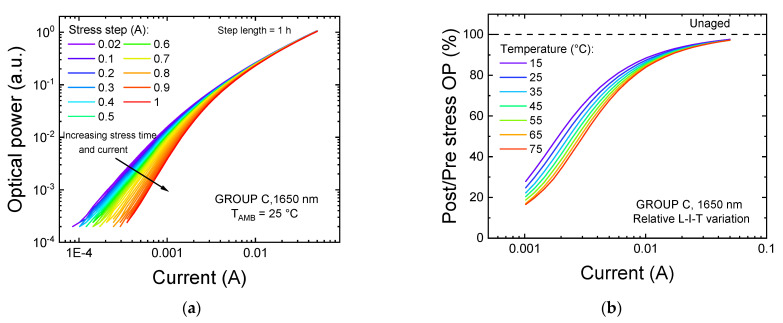
Variation of the optical characteristics of a group C sample during the step-stress procedure. (**a**) L–I characteristics measured after each stress cycle at T_AMB_ = 25 °C. (**b**) Relative variation of the L–I–T characteristic after the entire step-stress procedure. The dashed line represents the reference level, corresponding the optical emission of the unaged device at each measuring temperature.

To further prove this point, we analyzed the variations exhibited by the L–I characteristics of the device after each stress step, reported here in Figure 6a. While the variation in the OP at nominal current was within ±3% of its initial value, confirming the stability of the optical performance in this bias range, a total drop in emission in the low-bias regime up to 95% was found to affect all the DUTs. This variation is compatible with an increase in the SRH recombination rate, possibly induced by an increase in the concentration of non-radiative recombination centers within the active region of the device as a consequence of stress [23]. This hypothesis is further supported by the temperature dependence of the relative amount of degradation, reported in Figure 6b. In this plot, we can see that the worsening of the optical emission is stronger at higher measuring temperatures: this is again compatible with the increase in SRH recombination, because the SRH recombination coefficient *A* increases with increasing temperature [24]. 

### 3.2. Constant-Current Stress

In order to evaluate the acceleration of the degradation process under different bias regimes, a series of constant-current aging experiments were performed on the three groups of LEDs. Stress currents ranging from 0.35 up to 0.65 A were applied to the devices for total stress times up to 20,000 min, at an ambient temperature of T_STRESS_ = 45 °C. The stress was interrupted at logarithmic steps in order to evaluate the variation of the optical and electrical characteristics of the LEDs. 

**Figure 7:**
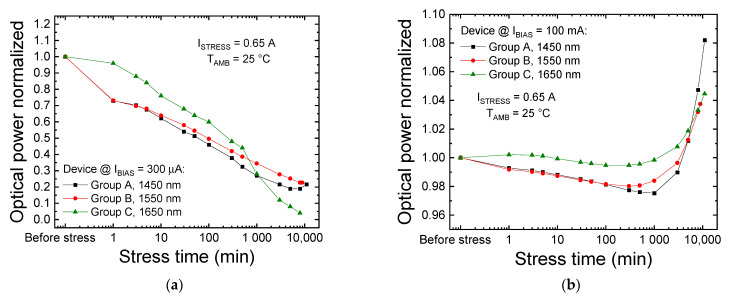
Trend of the optical power measured at T_AMB_ = 25 °C after each stress cycle for one representative LED of each group. (**a**) Optical power (OP) trend at 100 mA (nominal current) and (**b**) at 300 μA. Data are related to the constant-current stresses performed at T_STRESS_ = 45 °C.

Figure 7 shows the trends over time of the OP at 100 mA (Figure 7a) and at 300 μA (Figure 7b) during stress in the strongest condition, for I_STRESS_ = 0.65 A. All the samples under investigation showed similar variations: at high measuring current (100 mA, Figure 7a), the OP of the LEDs exhibited a slight initial decrease, less than 3%, followed by a recovery of the emission. At low measuring current levels (300 µA, Figure 7b), a strong drop in OP was detected for all the devices. This degradation process was found to be stronger in longer wavelength devices, up to 500 min of stress (at I_STRESS_ = 0.65 A). Above this aging time, a second degradation process seems to affect the optical emission of group C LEDs, which ended up showing the stronger amount of degradation during longer-term aging.

In the following paragraphs, both the high-bias and low-bias degradation processes will be discussed in detail. The three groups of LEDs exhibited similar degradation mechanisms, while differing in terms of degradation rate; therefore, the analysis will be focused on the data acquired from the aging experiments carried out on group C LEDs (similarly to what was done for the experimental data related to the step-stress procedure described in Section 3.1). This would also allow us to discuss the peculiar longer-term degradation process that was found to only impact the low-injection optical emission of this specific group of LEDs. 

#### 3.2.1. Variation in High-Current Regime

In high-bias regimes, the optical efficiency of an LED is typically determined by the Auger recombination rate, by the rate of escape and, in part, by SRH recombination. In addition, the presence of unwanted potential barriers may also limit the efficiency if the carrier injection is affected [25,26]. Most of these processes are activated by temperature, therefore the LED becomes less efficient if its junction temperature T_j_ increases as a consequence of stress. An increase in junction temperature can be either induced by a reduction in optical efficiency, possibly due to an increase in the rate of SRH recombination, or by the increase in power dissipation. This latter process is commonly induced by the increase in series resistance of the device.

**Figure 8:**
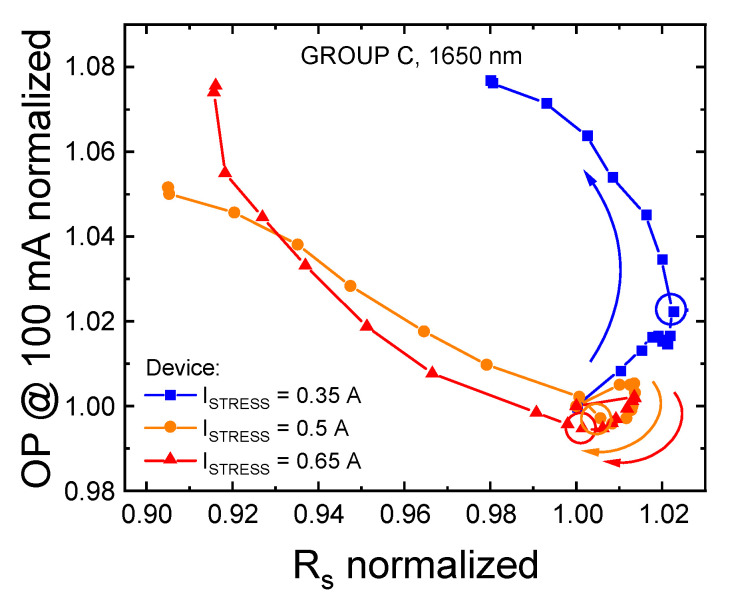
Correlation between the normalized series resistance and the OP measured at 100 mA (nominal current) and T_AMB_ = 25 °C after each stress cycle during the constant-current aging experiments carried out on group C samples, at the stress temperature of T_STRESS_ = 45 °C.

Figure 8 indicates that, as the recovery process begins dominating over the OP decrease, a correlation exists between the trend of the series resistance of the device and the variation in optical emission measured at 100 mA. This correlation cannot be ascribed to a reduction in power dissipation, because the measurements were carried out in a quasi-pulsed regime and the variation in power dissipation at the measuring current was minimal, below 1 mW. If we exclude self-heating effects and package-related modifications, the observed change in optical emission at high measuring currents may be attributed to variations in the local carrier density; these may be associated with a stress-induced reduction in current crowding, which results in a better spreading of current on the active area of the device [27]. This kind of modification may have originated from a current-induced annealing of the contact, or to a general improvement in the resistivity of the quasi-neutral regions induced by stress, for group C devices. This hypothesis is partially supported by that fact that group C devices exhibited a higher series resistance compared to groups A and B (see Section 2.2): the detected reduction in the series resistance of the third group of samples can therefore be explained as the progressive stress-induced improvement of the electrical characteristics of the ohmic contacts, whose processing was not as well optimized as for the other two families of LEDs under investigation. 

A second hypothesis on the origin of the OP recovery process takes into consideration the fact that although all the three groups of LEDs exhibited this peculiar variation during the last stages of stress, group A and B devices did not exhibit relevant variations in their series resistance. This may indicate that the OP increase process is not strongly correlated with the series resistance increase, and that this variation may just be related to a second physical process affecting the device characteristics during aging. Under this assumption, the optical recovery process can be explained by considering a modification of the injection efficiency induced by charge accumulation in proximity of the active region of the device [25,28]: defects with a non-neutral charge state that accumulate in specific device regions can induce a local variation in band bending, which may influence carrier transport, or their injection into the QWs if the accumulation occurs in proximity to the active region. This hypothesis is supported by the detected increase in defect density in proximity to the active region of all three groups of devices under investigation.

#### 3.2.2. Trend of the Non-Radiative Lifetime

In low-bias regimes, SRH recombination dominates over radiative recombination; therefore, the optical efficiency of the device strongly depends on the concentration of defects, present within the active region, which act as non-radiative recombination centers. In this section of the paper, we propose a methodology for analyzing the changes in the non-radiative recombination lifetime induced by stress, starting from optical characterization data. The proposed approach, albeit very simple, can be used to quantify the relative changes in SRH recombination induced by stress.

The figure of merit that describes the balance between radiative and non-radiative recombination within the QWs of an LED is the internal quantum efficiency (IQE), namely $\eta_{IQE}$, which is given by:

(2) $$\eta_{IQE}=\frac{\tau_{nr}}{\tau_{r}+\tau_{nr}}$$

where $\tau_{r}$ and $\tau_{nr}$ are the radiative and non-radiative carrier lifetimes, respectively. It is worth noting that this analysis is carried out by using optical data collected at very low currents (e.g., 500 µA). Such values correspond to low carrier densities, so Auger recombination is considered to be negligible for this analysis. Consequently, the efficiency of the devices is considered to depend only on the balance between radiative and SRH recombination.

Due to incomplete carrier injection from the electrodes into the QW, determined by the injection efficiency $\eta_{inj}$, and to non-ideal photon extraction, numerically determined by the extraction efficiency $\eta_{extr}$, only the so-called external quantum efficiency (EQE) $\eta_{EQE}$ of the device can be directly measured. This parameter is equal to the ratio of the rate of photons emitted by the device to the rate of carrier injected into the device, which are both measurable quantities. Therefore, the EQE can be written as

(3) $$\eta_{EQE}=\eta_{IQE}\eta_{inj}\eta_{extr}$$

This relationship describes a direct proportionality between the measurable EQE and the internal quantum efficiency of the device. Assuming that the stress is not impacting either on the extraction efficiency or on the injection efficiency, the relative rate of non-radiative recombination at time *t’*, which will be referred to as $\tau_{nr}^{\prime }=\tau_{nr}\left(t^{\prime }\right)$, with respect to time *t* can be expressed as:

(4) $$\frac{\tau_{nr}^{\prime }}{\tau_{nr}}\equiv \frac{\tau_{r}^{\prime }\eta_{IQE}^{\prime }}{1-\eta_{IQE}^{\prime }}\frac{1-\eta_{IQE}}{\tau_{r}\eta_{IQE}}≅\frac{\eta_{IQE}^{\prime }}{1-\eta_{IQE}^{\prime }}\frac{1-\eta_{IQE}}{\eta_{IQE}}\propto \frac{\eta_{EQE}^{\prime }}{1-\eta_{EQE}^{\prime }}\frac{1-\eta_{EQE}}{\eta_{EQE}}$$

where $\eta_{EQE}^{\prime }$ and $\eta_{IQE}^{\prime }$ are the external and the internal quantum efficiencies at time *t’*, respectively. The first equality of Equation (1) is maintained if we assume that the aging procedure does not impact on the radiative lifetime. Because $\tau_{r}\left(t\right)=1/Bn\left(t\right)$, this parameter depends both on the bimolecular recombination coefficient B, assumed to be constant and material- or epitaxy-specific, and on the carrier density n(t). For this reason, if we assume the carrier density at a specific bias level to be stable throughout the entire stress procedure, $\tau_{r}\left(t\right)$ will also be stable over time. As will be discussed in the following sections, in particular in Section 3.2.2, this assumption holds for groups A and B, whereas it may partially lose validity as the second degradation process start impacting on the optical characteristics of group C devices: in this case, Equation (4) underestimates the relative variation of the non-radiative lifetime.

With regard to the second approximation in Equation (4), considering that $\eta_{EQE}=\eta_{IQE}\eta_{inj}\eta_{extr}$, we assume that most of the variations related to the external quantum efficiency are due to stress-induced changes to the IQE, rather than to variations in the extraction or injection efficiencies. The former assumption is always valid when no package-related degradation [29,30] is observed; the latter point follows the considerations regarding the stability of the carrier density during stress. 

**Figure 9:**
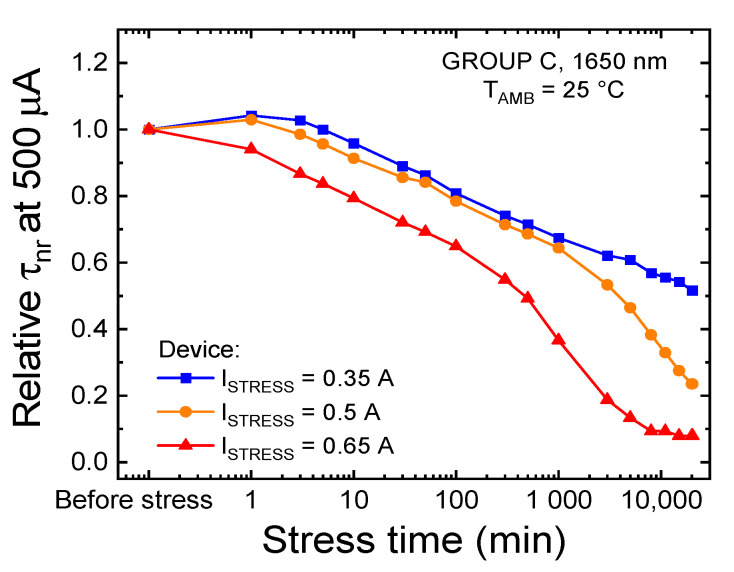
Relative trend of the non-radiative lifetime, extrapolated from the external quantum efficiency (EQE) calculated at 500 μA and T_AMB_ = 25 °C, during the three constant-current stresses carried out on group C LEDs at T_SRESS_ = 45 °C.

Figure 9 reports the relative variation of the non-radiative lifetime, calculated, on the basis of Equation (4), for the three constant-current stresses performed on the LEDs of group C. The experimental data indicate that: (i) the stress procedure induces an increase in the rate of non-radiative recombination, possibly due to the increase in the concentration of defects within the active region of the device; (ii) the degradation process is accelerated at higher stress current levels; and (iii) the onset of the second degradation process is observable at lower stress times for higher stress currents. These observations indicate that prolonged stress at injection levels in excess of 388 (722) A/cm^2^ induces the generation and/or propagation of non-radiative recombination centers (NRRCs) toward the active region of the device, in agreement with the hypotheses formulated during the analyses of the step-stress experiment data. At longer stress times, a second mechanism seems to be further reducing the radiative efficiency of the device: this aspect will be addressed in the following sections. 

#### 3.2.3. Emission in the Low-Bias Regime

In order to understand the physical origin of this second process, a more in-depth investigation of the variation in the low bias regime was carried out. 

**Figure 10:**
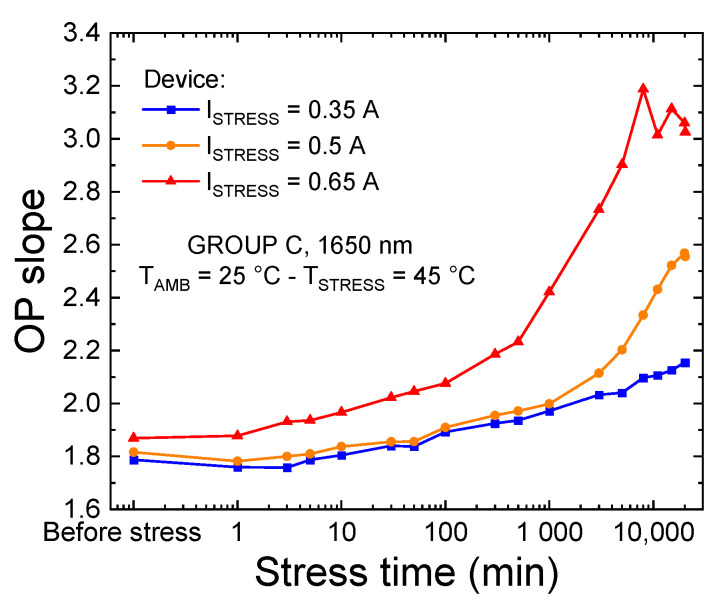
Slopes of the L–I curves, measured between 500 and 850 μA and T_AMB_ = 25 °C, during the three constant-current stresses carried out on group C LEDs at T_SRESS_ = 45 °C.

Further analyses of the trend of the OP in function stress time and current (Figure 6a) indicated that during aging, the LEDs exhibited a gradual increase in the slope of the log-log L–I curve under low-injection conditions (Figure 10). This increase was found to be stronger for devices aged at higher currents; additionally, it was found to be favored by the second degradation process that was identified for devices of group C.

Generally speaking, an ideal LED would have a log-log L–I curve with a slope equal to one, indicating a direct proportionality between the number of injected electrons and the number of emitted photons. Deviations from this trend indicate non-idealities, that may be related to non-radiative recombination processes (Auger, SRH, etc.), whose rate may change after degradation.

An increase in OP_slope_ toward values around two indicates that, within a specific bias range, SRH recombination is favored with respect to radiative processes [23]. Remarkably, for the highest stress current, the log-log slope of the optical power was found to increase to three (see Figure 10); such behavior is not common and deserves a more detailed investigation. To this aim, considering the fundamental rate equation based on the ABC model [31], we can write that:

(5) $$\frac{J\eta_{inj}}{qd}=An+Bn^{2}+Cn^{3}$$

where *J* is the injected current density, *q* is the electron charge, *d* is the thickness of the active region, *C* is the Auger recombination coefficient, and *n* is the carrier density within the active region. At low injection currents, SRH recombination dominates over radiative and Auger recombination processes, $An\gg Bn^{2}+Cn^{3}$, meaning that Equation (5) can be re-written as:

(6) $$\frac{J\eta_{inj}}{qd}≅An$$

In addition, at the end of the aging procedure at I_STRESS_ = 0.65 A, the OP_slope_ at low bias reaches values close to three, which indicates that the optical emission (L) becomes proportional to the third power of the injected current. From a mathematical perspective, this means that:

(7) $$L\propto Bn^{2}\propto J^{3}$$

If we combine this equation with the hypothesis of the SRH regime expressed by Equation (6), we obtain that:

(8) $$\{\begin{array}{c} \frac{J\eta_{inj}}{qd}≅An\\ L\propto Bn^{2}\propto J^{3} \end{array}\to \{\begin{array}{c} J\propto n\\ Bn^{2}\propto J^{3} \end{array}\to \{\begin{array}{c} J\propto n\\ J\propto n^{2/3} \end{array}$$

The last system reported in Equation (8) has no solutions, which indicates that one of the hypotheses upon which this system is based is wrong. Equation (7) mathematically represents an experimental evidence, and therefore must be right, which means that Equation (6) does not correctly define the physical behavior of the device, and a more detailed theoretical framework should be considered. By writing Equation (6), we were implicitly assuming no dependence of the injection efficiency on either the current density injected at the terminals or on the carrier density within the active region. In the low bias regime, the fraction of carriers that are injected into the quantum well (defined by the injection efficiency $\eta_{inj}$) can strongly depend on the presence of defects, because: (i) these defects can favor trap-assisted injection processes into the QWs; or (ii) carriers are lost outside the well through SRH recombination events [32]. Therefore, if we assume a dependence of $\eta_{inj}$ on current density, the system in Equation (8) can be rewritten as:

(9) $$\{\begin{array}{c} \frac{J\eta_{inj}\left(J\right)}{qd}≅An\\ L=Bn^{2}\propto J^{3} \end{array}\to \{\begin{array}{c} J\eta_{inj}\left(J\right)\propto n\\ Bn^{2}\propto J^{3} \end{array}\to \{\begin{array}{c} J\eta_{inj}\left(J\right)\propto n\\ n\propto J^{3/2} \end{array}\to \{\begin{array}{c} J\eta_{inj}\left(J\right)\propto J^{3/2}\\ n\propto J^{3/2} \end{array}$$

A solution of this set of equations can then be found by considering a square-root dependence of the injection efficiency on the injection current, that is: $\eta_{inj}\left(J\right)\propto J^{1/2}$. From a physical perspective, this finding indicates that, after stress, the injection efficiency monotonically increases with current, suggesting that the defect-assisted leakage paths are progressively saturated at increasing bias level [32]. Additionally, because a change in the injection dynamics has been detected for this specific set of samples, we must consider that Equation (4) may have limited accuracy once the second degradation process kicks-in. 

**Figure 11:**
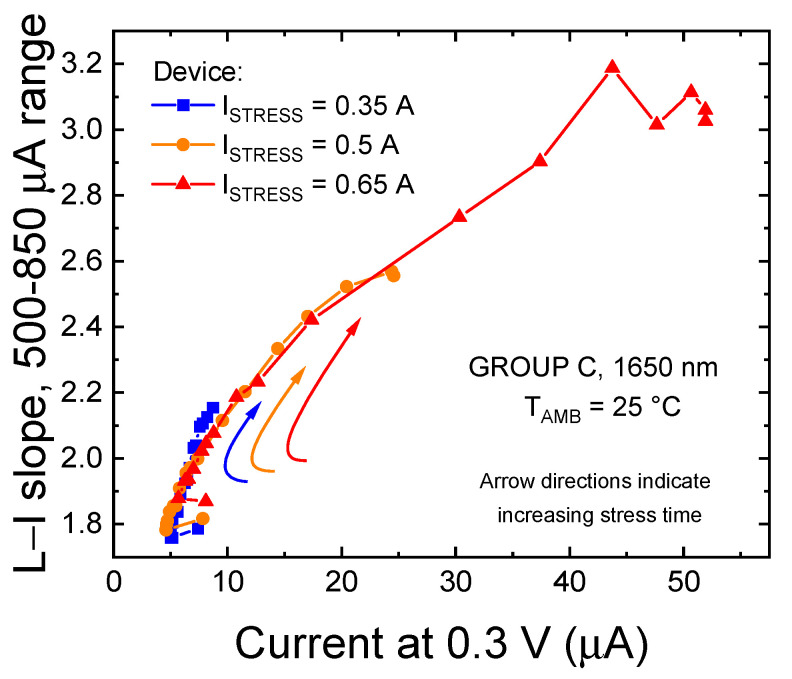
Correlation between the forward leakage current of the LED, measured at 0.3 V and T_AMB_ = 25 °C, and the slope of the L–I characteristics, calculated in the 500–850 μA range. Data related to the CC stress at T_SRESS_ = 45 °C on a group C device.

The increase in defect-assisted leakage paths as a consequence of stress was also testified by the correlation between the variation in the log-log OP_slope_ and the device current at low bias level, reported in Figure 11. At low voltages, current is strongly determined by defect-assisted conduction processes [22,33,34,35,36]; therefore, we can state that these defects are the main responsibility for the variation in optical emission observed in low bias regimes.

In addition, Figure 11 shows that a strong correlation between the absolute L–I slope and leakage current exists, even among different devices: this observation further supports our previous hypothesis and indicates that, for this particular family of devices, the optical and electrical characteristics, in bias regimes where conduction and recombination processes are assisted by defects, are strongly related. 

#### 3.2.4. Extrapolation of the Dominant Defect Parameters

From Figure 11, we can also observe that most of the optical and electrical degradation occurs after the onset of the second degradation process. In order to understand whether this stronger degradation should be ascribed to an acceleration of the defect generation process impacting on optical efficiency for shorter stress times, or if it should be ascribed to the propagation of a second kind of NRRC, we further investigated the role of trap level E_t_ in the variation of the non-radiative lifetime. 

To this aim, we defined a methodology for extrapolating the activation energy of the dominant SRH defect, starting from optical power vs. temperature measurements.

Under low injection conditions, assuming equal electron and hole densities, along with equal electron and hole cross-sections $\left(\sigma_{h}=\sigma_{e}=\sigma \right)$, the non-radiative lifetime associated with SRH recombination is approximately equal to:

(10) $$\tau_{nr}\left(T\right)=\tau_{0}\left[1+\cosh\frac{\Delta E}{kT}\right]$$

where $\tau_{0}={\left(N_{t}\sigma v_{th}\right)}^{-1}$, with thermal velocity $v_{th}=\sqrt{3kT/m_{e\left(h\right)}}$ and $m_{e\left(h\right)}$ being the electron (hole) effective mass; k is the Boltzmann constant and ΔE is the energy difference between the trap level $E_{t}$, calculated with respect to the conduction band, and the intrinsic Fermi level $E_{Fi}$ ($\Delta E=E_{t}$ − $E_{Fi}$). Considering Equation (10) and Equation (4), the ratio of the non-radiative lifetimes at temperatures T and T’ at a given time can then be expressed as:

(11) $$\frac{\tau_{nr}^{\prime }\left(T\right)}{\tau_{nr}\left(T\right)}=\sqrt{\frac{T}{T\prime }}\frac{1+\cosh\frac{\Delta E}{kT\prime }}{1+\cosh\frac{\Delta E}{kT}}\propto \frac{\eta_{EQE}^{\prime }\left(T\prime \right)}{1-\eta_{EQE}^{\prime }\left(T\prime \right)}\frac{1-\eta_{EQE}\left(T\right)}{\eta_{EQE}\left(T\right)}$$

**Figure 12:**
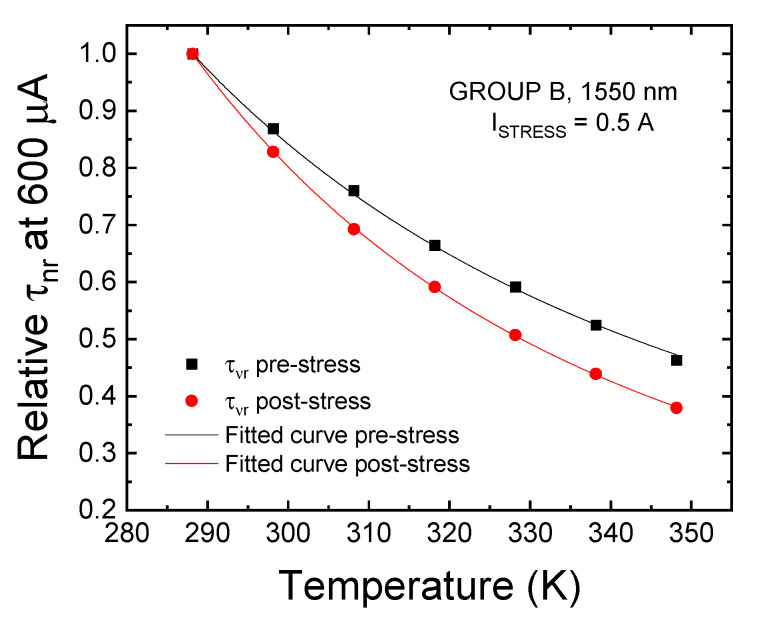
Relative trend of the Shockley-Read-Hall (SRH) lifetime with temperature, estimated before and after 9000 min of aging at 0.5 A for LEDs of group B.

Equation (11) indicates that the value of ΔE can be extrapolated from the relative variation of the non-radiative lifetime with temperature: this relationship can be derived through Equation (4), under the already mentioned assumptions, from experimental L–I–T data. As an example, Figure 12 reports the results of the fitting of the relative temperature trend of the non-radiative lifetime before and after stress on a representative sample of group B. 

The procedure described above was employed to estimate the level E_t_ of the dominant trap determining the SRH-related non-radiative lifetime under low-injection conditions. The results of this analysis are reported in Table 1.

The values of E_t_ reported in Table 1 were calculated considering reference E_fi_ levels of 0.428 eV (group A), 0.4 eV (group B) and 0.376 eV (group C), which correspond to the mid-gap of bulk semiconductors tuned for band-to-band emissions of 1450 nm, 1550 nm, and 1650 nm, respectively. Because we are dealing with QW devices, whose precise epitaxial structure is unknown, this assumption may lead to some errors in the estimation of the value of E_T_, which was found to be in the range of 510 ± 30 meV for the unaged devices. Nonetheless, the analysis holds if we analyze the variation in the estimated E_t_ after stress rather than its absolute value. From Table 1, we can see that while for group A and B devices the estimated E_t_ does not vary considerably after stress, heavily aged devices belonging to group C show a remarkable increase, up to +95 meV, in estimated E_T_ with respect to a fresh condition. This variation can be explained by considering that, as a consequence of the aging procedure, a new dominant NRRC is generated within, or propagated toward the active region of the device, thus further contributing to the reduction in the non-radiative lifetime associated to SRH recombination. This interpretation can also explain the onset of the second optical degradation process that was found to affect the optical efficiency in low-bias regime of heavily aged devices belonging to group C.

## 4. Conclusions

In summary, with this work we have extensively investigated the degradation mechanisms that can limit the reliability of InGaAs MQW LEDs tuned for emission in the 1.45–1.65 μm range. By submitting the devices to a series of accelerated aging experiments, we were able to ascribe the observed drop in optical efficiency at low bias levels to the decrease in the non-radiative lifetime, associated with the stress-induced generation/propagation of non-radiative recombination centers within the active region of the device. The experimental results indicate that for devices emitting at longer wavelengths, a second process contributes to speeding up optical degradation. 

To quantitatively describe the degradation processes, we defined two simple (although effective) methodologies: the first enables evaluation of the relative changes in SRH recombination lifetime induced by stress; the second permits estimations of the position of the dominant SRH defect relative to the mid-gap, thus providing information on the most relevant traps.

Through these methodologies, we were able to ascribe the second degradation mechanism detected on long-wavelength devices to the presence of a second non-radiative recombination center, which was also found to contribute to changes in the low-bias injection dynamics detected on heavily aged devices. The outcome of this work proves that the gradual defect-related degradation of modern InGaAs devices can still be an issue for their long-term reliability, and that a renewed research effort may be needed to ensure a longer lifespan to novel IR devices, even though those may be based on conventional and well-engineered III-V processes and epitaxies.

## Figures and Tables

**Table 1:** Estimated trap level values before and after 9000 min of constant-current (CC) stress at different bias levels. The measuring point chosen for the estimations was I = 600 μA.

	Before Stress		After Stress		
I_stress_ (A)	ΔE (eV)	E_t_ (eV)	ΔE (eV)	E_t_ (eV)	ΔE_t_^1^ (meV)
**Group A**					
0.5	0.119	0.547	0.108	0.536	−10.6
0.65	0.108	0.536	0.118	0.546	+10.3
**Group B**					
0.35	0.0826	0.483	0.122	0.522	+39.3
0.5	0.099	0.5	0.128	0.528	+28.24
0.65	0.097	0.497	0.127	0.527	+29.7
**Group C**					
0.35	0.152	0.527	0.212	0.588	+60.4
0.5	0.15	0.526	0.239	0.615	+89.4
0.65	0.156	0.532	0.252	0.628	+95.5

^1^—Variation in estimated E_t_ level after stress.

## Data Availability

Data sharing not applicable.
